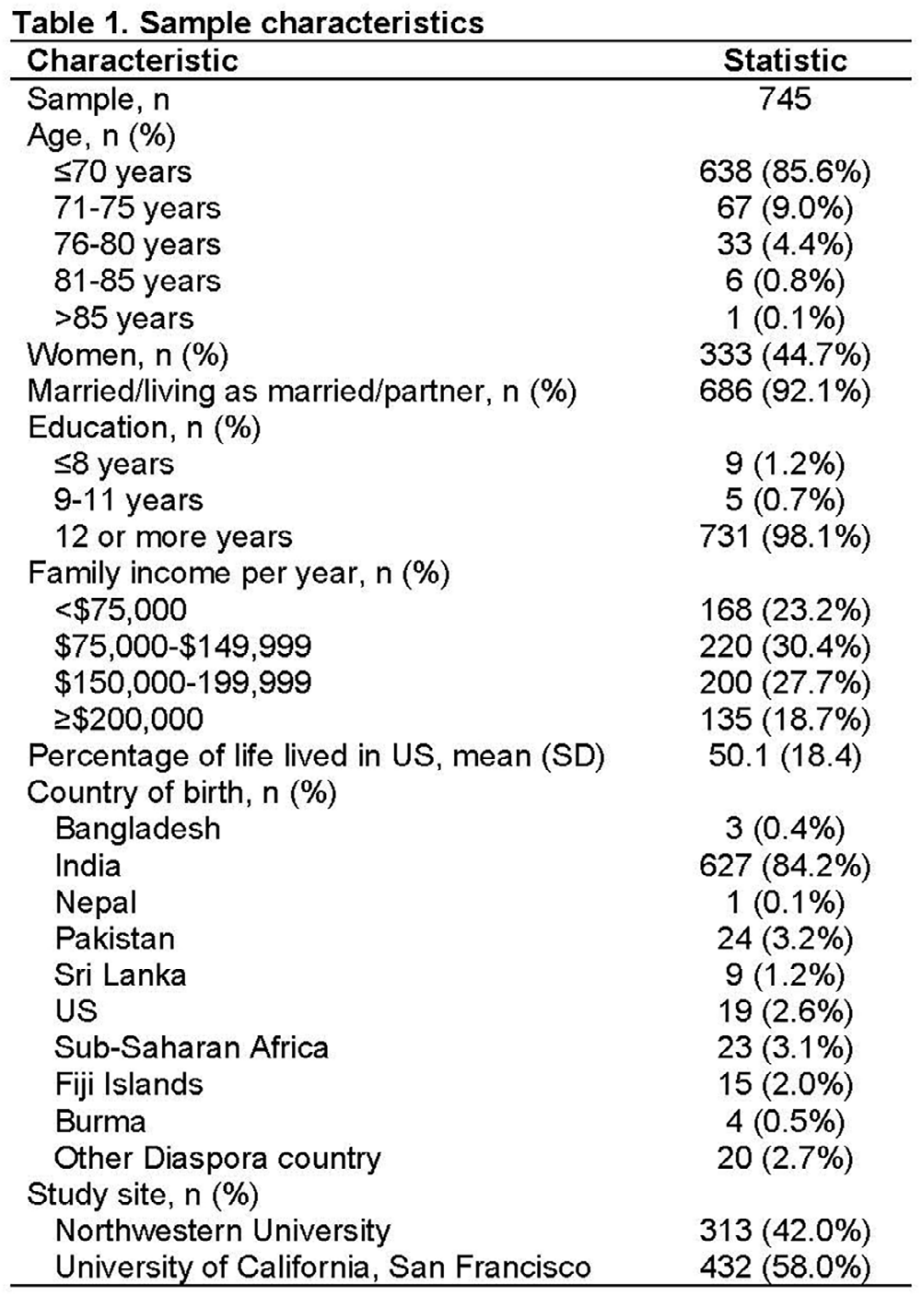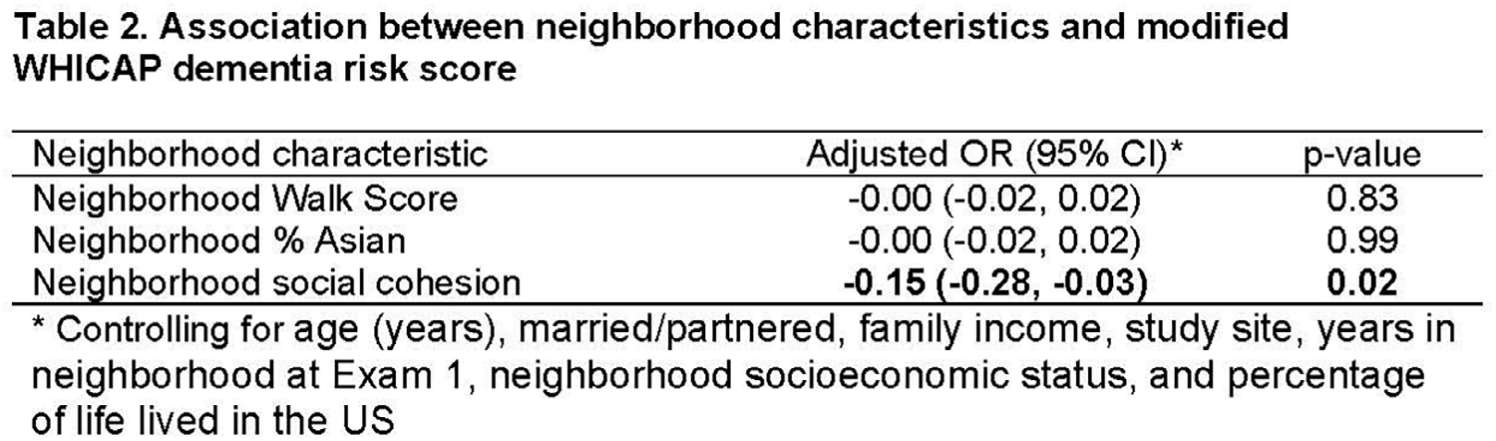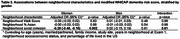# Neighborhood social cohesion, racial composition, and walkability and dementia risk: The Mediators of Atherosclerosis in South Asians Living in America (MASALA) Study

**DOI:** 10.1002/alz.085550

**Published:** 2025-01-09

**Authors:** Lilah M Besser, Preeti Pushpalata Zanwar, Oanh L. Meyer, Elisabeth K. Sohn, Irene H. Yen, Namratha R. Kandula, Alka M. Kanaya

**Affiliations:** ^1^ University of Miami Miller School of Medicine, Boca Raton, FL USA; ^2^ Jefferson College of Population Health, Philadelphia, PA USA; ^3^ University of California, Davis School of Medicine, Sacramento, CA USA; ^4^ The Center for Effective Philanthropy, San Francisco, CA USA; ^5^ University of California, Merced, Merced, CA USA; ^6^ Northwestern University, Chicago, IL USA; ^7^ Multi‐Ethnic Health Equity Research Center, University of California, San Francisco, San Francisco, CA USA

## Abstract

**Background:**

Prior studies suggest that neighborhood socioeconomic status, neighborhood walkability, and neighborhood social cohesion are associated with cognitive function and dementia risk. However, little is known about how neighborhood social and built environments influence dementia risk in South Asian populations residing in the US.

**Methods:**

We used data from 745 South Asian individuals ≥40 years in the US who completed Exam 2 (2015‐2018) of the Mediators of Atherosclerosis in South Asians Living in America (MASALA) Study. Neighborhood characteristics at Exam 1 (2010‐2013) included neighborhood percentage of Asian residents (range: 0‐87%), neighborhood Walk Score (i.e., walkability) (range: 0‐100), and neighborhood social cohesion (range: 5‐25). We calculated a modified Washington Heights/Inwood Columbia Aging Project (WHICAP) dementia risk score (range: 0‐52) for Exam 2 based on age, gender, education, diabetes, hypertension, current smoking status, low high‐density lipoprotein, and high waist‐to‐hip ratio. Multivariable linear regression examined associations between neighborhood characteristics and the dementia risk score, controlling for age (in years), marital/partner status, family income, study site, years residing in the neighborhood at Exam 1, neighborhood socioeconomic status, and percentage of life lived in the US. Interaction terms tested whether associations differed by gender.

**Results:**

Our sample was comprised of 45% women, 90% achieved a bachelor’s degree, and 84% were born in India, while the remainder originating from Pakistan, Sri Lanka, Sub‐Saharan Africa, the US, and other countries. Mean age was 59 years old (SD = 9). The mean dementia risk score was 8.4 for women (SD = 5.3) and 10.0 for men (SD = 4.8) (overall sample range: 0‐31). In multivariable analyses, greater neighborhood social cohesion at Exam 1 was associated with lower dementia risk scores at Exam 2 (estimate: ‐0.15, 95% CI: ‐0.28, ‐0.03). In stratified analysis, greater neighborhood social cohesion was associated with lower dementia risk scores for women (estimate: ‐0.28, 95% CI: ‐0.46, ‐0.10) but not men (estimate: 0.02, 95% CI: ‐0.15, 0.19) (interaction p‐value: 0.01). No associations were observed between neighborhood walkability or neighborhood percentage of Asian residents and dementia risk score.

**Conclusion:**

Neighborhood social cohesion may help reduce the risk of dementia among South Asian middle to older‐age women in the US.